# Role of GFI1 in Epigenetic Regulation of MDS and AML Pathogenesis: Mechanisms and Therapeutic Implications

**DOI:** 10.3389/fonc.2019.00824

**Published:** 2019-08-27

**Authors:** Tarik Möröy, Cyrus Khandanpour

**Affiliations:** ^1^Department of Hematopoiesis and Cancer, Institut de Recherches Cliniques de Montréal, Montreal, QC, Canada; ^2^Département de Microbiologie, Infectiologie et Immunologie, Université de Montréal, Montreal, QC, Canada; ^3^Division of Experimental Medicine, McGill University, Montreal, QC, Canada; ^4^Department of Medicine A, Hematology, Oncology and Pneumology, University Hospital Münster, Münster, Germany

**Keywords:** Gfi1, AML—acute myeloid leukemia, myelodyslastic syndromes, epigenetic abnormalities, DNA repair, metabolism

## Abstract

Growth factor independence 1 (GFI1) is a DNA binding zinc finger protein, which can mediate transcriptional repression mainly by recruiting histone-modifying enzymes to its target genes. GFI1 plays important roles in hematopoiesis, in particular by regulating both the function of hematopoietic stem- and precursor cells and differentiation along myeloid and lymphoid lineages. In recent years, a number of publications have provided evidence that GFI1 is involved in the pathogenesis of acute myeloid leukemia (AML), its proposed precursor, myelodysplastic syndrome (MDS), and possibly also in the progression from MDS to AML. For instance, expression levels of the *GFI1* gene correlate with patient survival and treatment response in both AML and MDS and can influence disease progression and maintenance in experimental animal models. Also, a non-synonymous single nucleotide polymorphism (SNP) of *GFI1, GFI1*-36N, which encodes a variant GFI1 protein with a decreased efficiency to act as a transcriptional repressor, was found to be a prognostic factor for the development of AML and MDS. Both the *GFI1*-36N variant as well as reduced expression of the *GFI1* gene lead to genome-wide epigenetic changes at sites where GFI1 occupies target gene promoters and enhancers. These epigenetic changes alter the response of leukemic cells to epigenetic drugs such as HDAC- or HAT inhibitors, indicating that *GFI1* expression levels and genetic variants of *GFI1* are of clinical relevance. Based on these and other findings, specific therapeutic approaches have been proposed to treat AML by targeting some of the epigenetic changes that occur as a consequence of GFI1 expression. Here, we will review the well-known role of Gfi1 as a transcription factor and describe the more recently discovered functions of GFI1 that are independent of DNA binding and how these might affect disease progression and the choice of epigenetic drugs for therapeutic regimens of AML and MDS.

## Myelodysplastic Syndrome and Acute Myeloid Leukemia

Myelodysplastic syndrome (MDS) can be considered a premalignant disease that mainly affects myeloid precursors. The course of this disease can vary from dependency on blood transfusions to a rapid progression toward acute myeloid leukemia (AML). A typical feature of MDS is an aberrant DNA hypermethylation at genes involved in apoptosis, differentiation, DNA repair and other functions, all of which are thought to be critical for the development of the disease, its progression and its therapeutic management (1). This postulate is supported by the beneficial effect on patients of treatment with low doses of DNA methyl transferase inhibitors such as the nucleoside analogs Azacitidine or Decitabine (2). By contrast, AML is a fully malignant, aggressive blood cancer characterized by massive accumulation of developmentally arrested (1, 2), immature blasts in the bone marrow (4–9). The prognosis for AML is based on cytogenetic findings and the spectrum of acquired genetic mutations. The generally used treatment approach includes the drug cytarabine (Ara-C, cytosine arabinoside), which is a compound similar deoxycytidine. Cytarabine is incorporated into replicating DNA strands, terminates strand elongation, and arrests cells in S-phase. Since it also inhibits DNA polymerase, double strand breaks occur which trigger replication checkpoints and ultimately cell death. The second substance used in induction therapy is doxorubicine, which intercalates between the bases of the DNA helix and also interferes with the action of the enzyme topoisomerase II, an enzyme that relaxes supercoiled DNA for transcription. As with cytarabine, the consequences are inhibition of DNA replication, DNA damage, alteration of the transcriptional program and also the eviction of histones from DNA (1, 3).

AML is the result of both genetic and epigenetic changes in genes involved in differentiation, proliferation, and epigenetic regulation. Overall, the prognosis for patients is poor although truly amazing progress has been made for the AML subtype with t(15;17) chromosomal translocations. This subtype, called acute promyelocytic leukemia (APL), carries a chromosomal translocation of the gene for the retinoic acid receptor alpha (RARA). APL is unique because it responds well to all transretinoic acid (ATRA) therapy, resulting in an excellent prognosis (4). Other subtypes, the so-called core binding factor AMLs (CBF-AML), typically feature t(8;21) or inv(16) translocations involving the *RUNX1, RUNX1T1*, and *CBFB* genes. Notably, the t(8;21) translocations results in the juxtaposition of the *RUNX1, RUNX1T1* genes (also called AML1 and ETO) leading to the expression of an AML1/ETO fusion protein, which can bind to DNA through the so called Runt Homology Domain (RHD) in the AML1 protein that is retained in the fusion. One of the target genes that is regulated by AML1/ETO is *GFI1* (5–7). GFI1, itself, can interact with the AML1/ETO fusion protein, since it binds to the Nervy homology region 2 (NHR2) in the ETO protein. This protein domain has been shown to be critical in the function of the AML1/ETO fusion protein for induction of AML (8, 9).

Up to 90% of patients with CBF-AML respond well to treatment although relapses occur in roughly half of these (1, 3). From the remaining AML patient cohorts, overall only less than half of those younger than 60 years can be cured despite aggressive therapeutic approaches. The success rate is even lower for those older than 60 years who cannot tolerate the harsh therapeutic approaches required and <20% survive more than 5 years (10). Hence, new therapeutic approaches are urgently needed to improve prognosis.

Significant advances have been made in the understanding of AML induction and progression at the molecular level. As a result, a series of new drugs, such as inhibitors of epigenetic modification of DNA or histone or other therapeutic strategies that go beyond classical chemotherapy have been evaluated, but without a therapeutic breakthrough (11). One reason for this could be that the activation of many pathways that confer resistance to treatment occurs during relapse or as a direct consequence of chemotherapy. Also, gene expression profiling and genomic sequencing of leukemic cells from AML patients have revealed a large degree of heterogeneity in the leukemic cell population, in particular with regard to the acquired genetic mutations (12). As a consequence, AML cells may have a variety of pathways through which they can escape the broad and non-specific action of the classical radiation- and chemotherapy.

## Growth Factor Independence 1—GFI1

### GFI1 as a Transcription Factor

**Figure 1:**
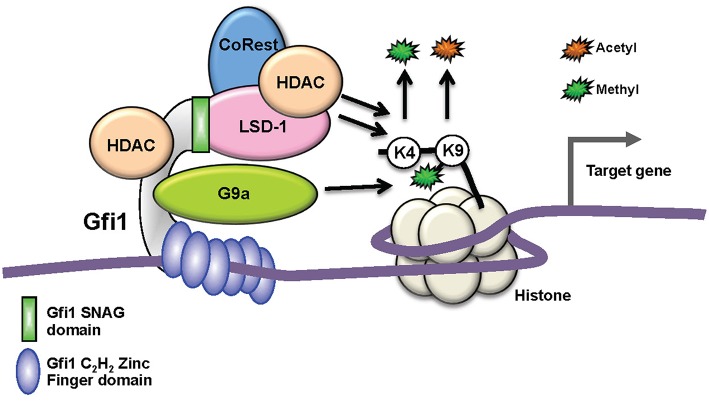
Biochemical function of GFI1 as a transcription factors. Schematic depiction of GFI1 in a complex with its interacting proteins occupying a genomic site 5′ of a target gene. Whereas, the recruitment of the LSD1/CoRest complex occurs through the N-terminal SNAG domain (green), the middle domain (gray) serves mainly as an interaction platform for other proteins and the C_2_H_2_ zinc finger domains (blue) mediate DNA binding. G9A and HDACs can bind directly to GFI1, but HDACs can also be part of the LSD1/CoRest complex. HDACs and LSD recruitment leads to the removal of acetyl- or methyl groups from Histone H3 lysine 9 or 4, respectively. G9A enables the methylation of H3 lysine 9.

The development of both hematopoietic and leukemic cells is regulated to a large extent by transcription factors (TFs) that determine lineage specificity, differentiation, and cell proliferation and these represent endpoints of receptor-initiated signaling pathways that drive and control leukemogenesis (13, 14). As a consequence, the deregulation of many transcription factors (15–19), can directly induce malignant transformation, such as is seen with the loss of the transcription factor and tumor suppressor TP53 (p53). GFI1 is another such transcription factor that plays a critical role in both myeloid differentiation and in the development of AML. GFI1 is a nuclear protein with three identifiable domains: (i) an N-terminal 20 amino acid “SNAG” repressor domain, which is shared between GFI1 and the transcription factors SNAIL and SLUG, (ii) six highly conserved C-terminal zinc-finger domains, and (iii) an intermediate domain that separates the SNAG and zinc finger domains, which is not well-conserved across species (Figure 1). Zinc fingers 3–5 of GFI1 are critical for binding to a specific DNA sequence motif [taAATCac(t/a) gca], whereas zinc fingers 1, 2, and 6 can also mediate interactions with other proteins [reviewed in (20–22)]. Typical target genes of GFI1 include *Hoxa9, Pbx1*, and *Meis1* or the *CSF1* and *CSFR1* genes, which play important roles in myeloid differentiation (21).

**Figure 2:**
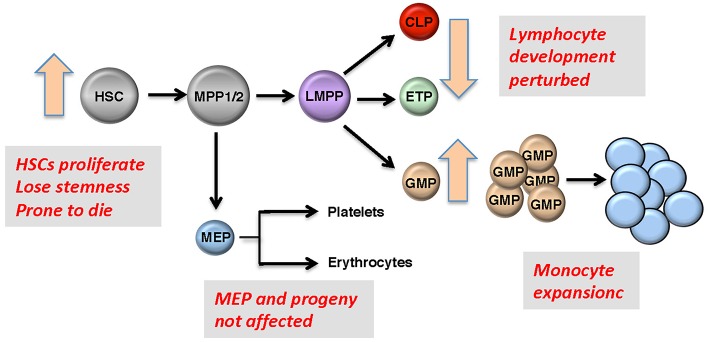
Roles of GFI1 in hematopoiesis. Representation of early hematopoietic differentiation; HSC, hematopoietic stem cells; MPP, multipotent progenitors; LMPP, lymphoid-primed multipotent progenitors; CMP, common myeloid progenitor; GMP, granulocyte-monocyte progenitor; MEP, megakaryocyte erythroid progenitor. Boxes: GFI1 deficiency leads to defects in HSCs, GMPs, and in early T- and B-precursors, but MEPs and their progeny are not affected since Gfi1 is not expressed in these cells. One of the most prominent features of GFI1 deficiency is the accumulation of myeloid cells that are at an early stage of differentiation.

GFI1 is expressed in hematopoietic stem cells (HSCs), in lymphoid and myeloid precursors, and during early steps of B- and T-cell differentiation. Expression ceases in mature lymphoid cells and macrophages, but is reactivated upon receptor-mediated stimulation in all these cells. Moreover, GFI1 expression has been detected in dendritic cells and the so-called “type 2 innate lymphoid cells” (ILC2), which control parasitic infections and allergic reactions (23). Ablation of Gfi1 in mice affects the function of all cells in which it is expressed and perturbs myeloid development, activation of macrophages, differentiation, activation of T- and B-lymphocytes, the proliferation, and self-renewal of HSCs as well as the function of dendritic cells and ILC2 cells (24–27). Hence, with the exception of the erythroid/megakaryocyte lineage, GFI1 exerts control over not only the majority of early hematopoiesis, but it also regulates the function and reactions of mature effector cells of both the acquired and innate immune system (28, 29) (Figure 2). Our knowledge to-date on GFI1 suggests that it exerts these functions through its canonical action as a transcriptional repressor, which is mediated by recruiting histone-modifying enzymes to its target genes (Figure 1) (22). In this role GFI1 mainly interacts with histone deacetylases (HDACs; HDAC-1, −2, and −3), the histone methyl transferases EHMT2 (G9A) or the histone demethylases KDM1A (LSD1) (8, 30, 31), and recruits these enzymes to target genes harboring the GFI1 binding sequence. This leads to deacetylation of lysine 9 of histone 3 (H3K9) followed by di-methylation of H3K9 or de-methylation of H3K4 (histone 3, lysine 4) at these sites and consequently to gene silencing (20–22, 30).

### GFI1 in DNA Repair and the Regulation of TP53; A Non-canonical Function

DNA repair has attracted considerable attention in recent years because of its proposed contribution to therapy resistance during chemotherapy (CTX) (32). Because a number of chemotherapeutic drugs (including Ara-C, doxycycline, and others used in the treatment of AML) induce DNA double strand breaks leading to cell death, efficient repair of these breaks could reduce the effectiveness of chemotherapy using these agents. The recent observation that GFI1 can facilitate DNA repair comes from unexpected results obtained while analyzing the GFI1 related proteome in human cells (33). While both immune-precipitation and Bio-ID experiments revealed the expected histone-modifying enzymes and transcriptional co-factors as binding partners for GFI1, a number of other proteins related to DNA repair were also found to bind to GFI1, including the DNA repair factors MRE11 and TP53BP1 as well as the arginine methyl transferase PRMT1. MRE11 is a member of the MRN complex that includes MRE11, NBS (Nijmegen Breakage Syndrome), and RAD50 (34–36). This complex binds to double strand DNA breaks and initiates a number of steps leading to DNA repair by both non-homologous end joining (NHEJ) and homologous recombination (34–36). TP53BP1 is one of the main regulators of DNA double strand break repair and it helps determine whether NHEJ or homologous recombination (HR) is used for the repair (34–36). How exactly TP53BP1 enables this choice is unclear, but during the G1 phase it acts specifically on NHEJ. PRMT1 catalyzes arginine methylation of histones leading to either activation or repression of transcription, but it also has many other non-histone substrates, which are involved in a large variety of biological processes (37). Among these non-histone substrates are MRE11 and TP53BP1 and PRMT1 is responsible for an asymmetric methylation of arginine in MRE11 that activates its ability to repair DNA (38).

Biochemical tests have confirmed that GFI1 forms a tripartite complex with PRMT1 and either MRE11 or TP53BP1 and enables the methylation of these proteins at specific sites thus rendering them active. GFI1 deficiency leads to under-methylated MRE11 and TP53BP1 and subsequently to deficits in DNA repair (33). This is intriguing for several reasons: first this is a function of GFI1 that is independent of DNA binding and thus entirely different from its canonical function as a DNA-binding transcriptional repressor. This is not the only non-canonical mode of action of GFI1; we have previously shown that GFI1 can recruit LSD1 not only to promoters of target genes where it modifies histone H3K4, but also to the tumor suppressor protein p53 to remove methyl groups from its C-terminal domain (39). This interaction between GFI1 and p53 also occurs in the absence of DNA binding, similar to its interaction with MRE11 and PRMT1 (31, 40). The C-terminal domain of p53 contains a number of lysine residues that can be modified post-translationally, such as by methylation (39) and the effect of methylation is generally considered to dampen or control p53 activity. Thus, the recruitment of LSD1 to p53 by GFI1 would render p53 less active through a decrease in active lysine methylation (40, 41). Conversely, GFI1 deficiency leads to a hypermethylation of p53 and causes an over-activation of p53 target genes and subsequently p53-mediated cell death (40). It may therefore be of interest to engineer ways to inhibit GFI1 as a means to develop new, targeted AML therapy. This would have two effects: first, the inhibition of DNA repair which should potentiate the DNA-damaging effect of drugs or radiation and second, the activation of p53 to drive leukemic cells into apoptosis. However, experiments that engineered reduced expression of GFI1 as well as the study of cells with defective *GFI1* alleles have indicated that decreasing the action of GFI1 can accelerate myeloid leukemogenesis (42). Hence, only a complete abrogation of *GFI1* expression or efficient destruction of the GFI1 protein may work in this strategy. A new generation of drugs that target specific proteins for ubiquitin mediated degradation could be one solution to this challenge.

## GFI1 and AML

Evidence for a role of GFI1 in AML emerged almost a decade ago when studies first indicated that the *GFI1* gene was expressed in several human myeloid leukemia cell lines as well as cells from patients with different types of myeloid leukemia including AML (21, 43, 44). A functional link between GFI1 and AML was suggested by the observation that a coding single nucleotide polymorphism (SNP) in the human *GFI1* gene (rs34631763) was associated with AML (45). The protein encoded by the allele harboring this SNP contains an asparagine in position 36 instead of the normally occurring serine of GFI1 (GFI136S), and was therefore named the 36N (*GFI1*36N) variant. This amino acid exchange falls into the middle domain that separates the SNAG domain from the zinc fingers and may therefore be important for protein-protein interactions. Indeed, in contrast to the more frequent form, the 36N variant does not co-localize and bind to the RUNX1/RUNX1T1 (AML1/ETO) fusion protein found in AML patients with a t(8;21) translocation. Also, GFI1 expression was found to be increased in samples from t(8;21) patients as compared to those patients that did not carry this rearrangement. Similarly, a correlation between GFI1 expression with a higher incidence of mutations in *NPM1* and *FLT3-ITD* and *KTM2A* rearrangements was observed (46). Although this indicated a role of GFI1 and its 36N variant in this subtype of AML, a clear correlation with overall survival of the respective patients remains to be shown with larger cohorts of t(8;21) patients. However, the presence of the *GFI1*-36N allele is clearly correlated with an increased risk of MDS patients to develop an AML (47).

**Figure 3:**
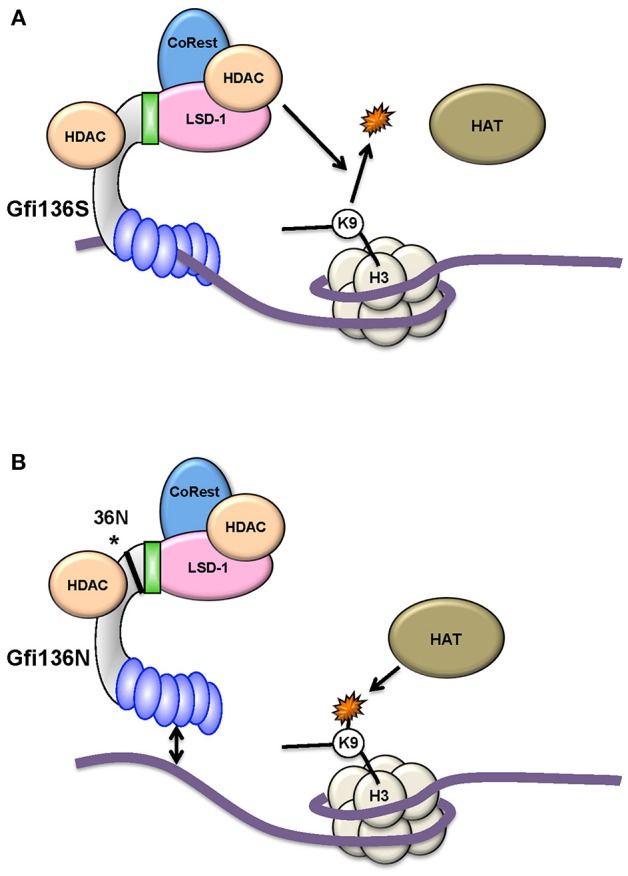
The GFI136N variant affects epigenetic modifications. The more common GFI136S variant **(A)** forms more stable complexes with chromatin than the variant GFI136N, which is associated with AML **(B)**. As a consequence, in cells carrying the GFI136N variant (amino acid change is marked with an asterisk*), the recruitment of HDACs is less efficient at GFI1 target genes and H3K9 acetylated forms accumulate due to HAT activity. In this situation, a HAT inhibitor is more effective as an epigenetic treatment for AML than a HDAC inhibitor [from Botezatu et al. (49)].

Studies with knock-in (KI) mice (generated by gene targeting) that carry either the human *GFI136N* variant allele or the more common human *GFI136S* allele at the endogenous murine *Gfi1* locus (13) provided more insight into the mechanism underlying the association of GFI1 with MDS and AML. The presence of the *GFI136N* variant allele led to a proliferative expansion of myeloid precursors such as GMPs (granulocyte-monocyte progenitors) similar to a complete GFI1 knockout (48). In addition, the GFI136N protein binds less stably to target gene promoters than the 36S form. Experiments with 36N and 36S KI mice showed that cells expressing only the GFI136N variant are unable to efficiently de-methylate lysine 4 of histone H3 (H3K4) or de-acetylate lysine 9 of histone H3 (H3K9) near *Gfi1* target genes such as the *Hoxa9* promoter (Figure 3). These findings were confirmed using cells from patients carrying either two GFI136S alleles or one GFI136S and one GFI136N allele (49). It can be concluded that expression of GFI136N changes epigenetic marks genome-wide at *Gfi1* target sites and, as a consequence, the presence of the GFI136N protein leads to higher levels of *Gfi1* effectors such as *HoxA9* expression (17, 48, 49). Indeed, AML patients carrying the *GFI1* variant allele have relatively high levels of HOXA9 (48), an effect which may contribute to the progression of the disease. Support for this concept comes from experiments which show that GFI136N accelerates myeloproliferative disease initiated by a mutant *KRAS* gene (48). It has therefore been proposed that the GFI136N variant correlates with a preleukemic state in myeloid precursors facilitating the development of an AML (17, 48, 49).

Further studies with 36N and 36S KI mice and other mouse models that carry a low-level expressing *GFI1* allele (*GFI1* expression knockdown or *GFI1*-KD animals) showed that either the presence of the *GFI136N* allele or reduced expression of the *GFI1* gene accelerated both the initiation and progression of AML in mouse models in which myeloid leukemogenesis is driven by expression of an onco-fusion protein (17). In all mouse models carrying a *GFI136N* KI gene, the presence of one allele of *GFI136N* was sufficient to induce acceleration of leukemogenesis, pointing to a dominant effect of the GFI136N protein. This resembles the situation in human patients, since most of *GFI136N*-expressing AML patients are heterozygous for *GFI136N* (17, 45, 48, 49).

Leukemic cells from GFI1-KD mice expressing onco-fusion proteins showed an increase in H3K9 acetylation at GFI1 target genes which would be expected considering that lower levels of GFI1 would be less efficient to recruit HDACs to target gene promoter sites (17, 48, 49). Similarly, the presence of the *GFI136N* KI allele was also accompanied by higher levels of H3K9 acetylation in leukemic cells from mice, supporting the notion that the *GFI136N* allele encodes a deficient form of GFI1 or one which is less able than the more common GFI136S form to exert its function as a transcriptional repressor (17, 45, 48, 49). One reason for this could be the lower DNA binding ability of the GFI136N variant compared to the more common GFI136S form, consistent with observations from chromatin immune precipitation (Ch-IP) experiments (17, 45, 48, 49). It is conceivable that with a lower level of GFI1 at target promoter sites the recruitment of HDACs will be also less efficient, leading to less efficient deacetylation of H3K9 residues. Interestingly, and possibly as a consequence, leukemic cells from both mouse models (*GFI136N* and *GFI1*-KD) were more responsive to histone acetyltransferase inhibitors (HATis) than to histone deacetylase inhibitors (HDACis), which are more typically used in experimental therapies for AML (17, 49). Although more in-depth investigation is warranted, these studies point to the possibility that *GFI1* expression levels or the presence of a variant allele could be biomarkers for disease outcome and informative on the choice of epigenetic therapy.

## GFI1 and MDS

Loss of *Gfi1* in mice can lead to a significant accumulation of myeloid precursors (Figure 2), that also appear in the peripheral blood and secondary lymphoid organs, a phenotype that is even more pronounced when apoptosis is inhibited by co-expression of a *BCL2* transgene in a *Gfi1* knockout background (10, 11). This situation is reminiscent of a myeloproliferative disorder or a precursor stage to AML with increased numbers of blast cells, similar to what is observed in patients with myeloproliferative neoplasms (MPN) or MDS. Low levels of GFI1 in the blasts of MDS patients have also been reported to increase the probability of progression of MDS into AML and correlate overall with an inferior prognosis (17, 45, 48, 49). One mechanism how GFI1 might be downregulated in AML and MDS patients is through deletion of parts of the 1p chromosome, on which the *GFI1* locus is localized. In one study, the *GFI1* locus was the common locus deleted among different patients with deletion of the 1p chromosome (17). This is corroborated by studies in mice with reduced expression levels of GFI1 which results in a myeloproliferative disease that is not only fatal, but also resembles MDS and is a rather common to MPN in many aspects since it progressed to an overt leukemia when additional mutations were induced either by retroviral infection or chemical carcinogenesis (42). That study concluded that reduced Gfi1 levels represent a high risk for development of a myeloid leukemia, since the low Gfi1 levels lead to the accumulation of myeloid cells like in a full Gfi1-deficient animal, but a p53 mediated cell death is not activated in GFI1-KD cells as it is in *Gfi1*-deficient cells (42). As a result, these cells can continue to accumulate and be transformed into leukemic cells. Additional reports showed that the variant *GFI136N* form, which had been reported to be associated with AML, also predisposes patients to MDS development, and presence of this variant was an adverse prognostic factor after adjusting for age, sex, bone marrow blast count, cytogenetic findings, and IPSS score (47).

Similar to AML, the frequency of the variant *GFI136N* allele was significantly increased compared to that of the *GFI136S* allele in MDS patients from several cohorts compared to healthy controls (17, 45, 48, 49). The data indicated that the presence of this variant was an independent adverse prognostic factor for the overall survival of the patients, even when taking into account the progression to AML as well as leukemia-free and event-free survival. The study also reported that *GFI136N* patients had a poor response to azacitidine, a hypomethylating drug used to treat MDS patients (17, 45, 48, 49). The data indicated that among all MDS patients treated, those carrying a *GFI136N* allele had a worse outcome than those carrying the more common *GFI136S* allele. Hence, as for AML, both GFI1 expression levels as well as the presence of a “hypomorphic” *GFI1* allele have prognostic value and may also be indicative for the success of the specific epigenetic therapy used in MDS (47, 49).

## The Role of the GFI1 paralogue GFI1B in AML and MDS

Growth factor independence 1b (GFI1B) is a protein with high similarity to GFI1 and both the N-terminal SNAG domain and the C-terminal zinc finger domains are almost identical in their amino acid sequence to those found in GFI1. However, the middle part that separates these two domains is not conserved and the sequences bear no similarity at all. Also, GFI1B is encoded by a different gene located on another chromosome. The high sequence similarity in their SNAG- and zinc finger domains suggests that they may have originated by gene duplication. Functionally, GFI1B is essential not only for the regulation of HSC dormancy and proliferation (50) but also for erythroid and megakaryocytic differentiation and the generation of platelets (51–58). Moreover, GFI1B is also involved in the development of B- and T-cells (20, 59–61). Similar to GFI1, GFI1B can recruit histone-modifying enzymes such as the KDM1A/RCOR1 (LSD1/CoREST) complex, the methyltransferase G9a, or HDAC to gene promoters (8, 30, 62, 63). The p38 MAPK pathway has also been proposed as one of the GFI1B downstream effectors in the development of erythrocytes and platelets (64). More recently, it has been shown to play a role as a regulatory factor in the Wnt signaling pathway by forming a tripartite complex with beta-catenin and LSD1 at regulatory sites of Wnt effector genes (65).

Despite their similarities, loss of either *Gfi1* or *Gfi1b* leads to very different effects on hematopoietic cells. Constitutive deletion of *Gfi1b* in mice results in embryonic lethality around day E14.5 due to a failure in erythrocyte and megakaryocyte development (55, 61). Conditional loss of Gfi1b in adult mice leads to a significant expansion of functional HSCs in the BM and peripheral blood (PB) (50). The group of Reijden also reported a mutation of the zinc finger 5 of GFI1b leading to a truncated protein missing the domain responsible for DNA binding. This mutation acts in a dominant negative manner and leads to establishment of a Gray Platelet syndrome (66).

In contrast, constitutive deletion of Gfi1 leads not only to a complete loss of HSCs' stemness (27), but also to a severe neutropenia accompanied by accumulation of immature monocytic cells both in the BM and PB (29), suggesting that GFI1 and GFI1B may function biochemically in a similar way, but since they exert these functions in different cell types, have also very different biological roles.

Evidence that the GFI1B expression level may be linked to leukemia comes from experiments showing that forced expression of GFI1B inhibited IL-6-induced cell cycle arrest and differentiation in the murine myeloblastic leukemia cell line M1 (67) and resulted in expansion of immature erythroblasts and repression of myeloid in human primary hematopoietic progenitors (57). Moreover, *GFI1B* expression was found to be lower in CD34^+^ leukemic cells derived from AML or MDS patients as compared to CD34 positive BM cells from healthy controls (68). In addition, reduced *GFI1B* expression in blast cells was associated with inferior outcome with regard to overall survival (OS) and event free survival (EFS) of both MDS and AML patients from different cohorts (69, 70). It has been reported that loss of one allele of *Gfi1b* accelerates AML development in different transgenic mouse models and that this effect was even stronger when both alleles of *Gfi1b* were deleted. The study showed that the number of leukemic stem cells (LCSs) was significantly increased upon loss of *Gfi1b* and was most likely responsible for driving leukemia development (68). Similar to the situation with *Gfi1*, loss of *Gfi1b* led to genome-wide changes, which correlated with accelerated AML development. However, in the case of *Gfi1b*, changes in the p38 (MAPK14)/AKT/FOXO signaling cascade were observed in agreement with other reports linking GFI1B expression to p38 MAPK activation.

Besides alteration in the level of GFI1b, Anguita and colleagues elegantly demonstrated that mutation in zinc finger 4 of GFI1b promote AML development by disturbing the SPI1/GFI1/GFI1b regulatory network (71).

So far, these reports suggest that GFI1 and GFI1B are both implicated in MDS and AML through distinct molecular mechanisms and different biological functions, although for both factors their expression level is critical. Reduced expression or loss of GFI1B affects HSCs and LCSs and accelerates leukemogenesis, possibly through regulation of Wnt signaling, but also through epigenetic changes such as increased H3K9 acetylation levels and alteration of the ROS/p38/Akt/FoXO signaling cascade. In contrast, reduced expression of GFI1 or its reduced ability to bind target genes does not have the same effect. Here, myeloid precursors are affected and undergo an expansion, which can give rise to a full myeloid leukemia in mice, but only if accompanied by additional mutations. The underlying mechanisms include an epigenetic component such as an increase H3K9 acetylation as in the case of GFI1B, but in addition involve metabolic stress and the regulation of p53 through LSD1/GFI. However, for both GFI1 and GFI1B a reduced expression or function is associated with an inferior prognosis of AML patients and in the case of GFI1 with a faster progression of MDS to AML.

## GFI1 Status and Epigenetic Drugs to Treat AML

### GFI1 Status and Expression Levels Affect Histone Acetylation

One of the most promising avenues to improve AML outcome is the search for drugs that affect epigenetic mechanisms of gene regulation. The concept behind this strategy is that blocking histone deacetylation will maintain chromatin in an open conformation and promote active gene expression and thus enable leukemic cells to differentiate into more mature stages that are postmitotic and more readily eliminated by classical chemotherapy (11, 72). A number of drugs that target histone-modifying enzymes such as inhibitors of HDACs, have been used in clinical trials with mixed results indicating their limited therapeutic effect (72). One reason behind this lack of overt success could be that particular leukemia subtypes require specific inhibitors. Evidence in support of this possibility comes from studies with the *GFI136N* variant. Analysis of genome-wide histone methylation and acetylation patterns in leukemic cells from AML patients or from mice which carry either the *GFI136S* or *GFI136N* allele revealed some interesting differences. Cells in which *GFI136N* was expressed showed increased acetylation levels of histone 3 at lysine 9 (H3K9ac) at GFI1 target genes as compared to *GFI136S-expressing cells*, and such modifications are associated with active transcription (47, 49). The affected genes were found to be involved in roles such as cell proliferation and transcription in both human and murine AML samples. Leukemic cells from mice and from human patients were treated with inhibitors of HDACis and histone acetyl transferase (HATis). The results showed that in both cases, leukemic cells with a *GFI136N* allele or with reduced *GFI1* expression levels were more sensitive to HATis than to HDACis, which are more typically used than HATis in experimental therapies. These studies point to the possibility that that *GFI1* expression levels or the presence of the *36N* variant allele could be biomarkers for disease outcome and informative on the choice of epigenetic therapy.

Other studies revealed that AML patients with reduced *GFI1* expression levels showed increased levels of heme-oxygenase (HO-1; an enzyme involved in heme metabolism), HDAC1, HDAC2, and HDAC3 in correlation with poor prognosis (73). Cells from these patients showed resistance to the induction of apoptosis by Panobinostat, a non-selective pan-HDAC inhibitor (73). A link between GFI1 protein levels and AML development and prognosis was further corroborated by a study in which GFI1 was overexpressed in leukemic cell lines and primary murine cells. This led to growth inhibition and a reduced potential for colony formation (74). In addition, high Gfi1 levels impaired expansion of pre-leukemic cells in the mouse and in a humanized model caused myeloid differentiation and a decrease of precursor numbers (74). However, other studies using data from AML patients with a normal karyotype observed poorer survival rates when *GFI1* expression levels were increased (75). Hence, whether altering GFI1 levels could be a new therapeutic approach for AML remains an open question that will require additional experimental evidence for resolution. Nevertheless, the findings from all of these studies indicate that GFI1 status or expression level may be indicative of prognosis and disease progression and may also inform which epigenetic-targeting drug should be used in a therapeutic regimen. However, it is also clear that these observations are context-dependent, suggesting that personalized genomic profiles and more detailed classification of AML patients into genetically defined subgroups that are either more sensitive or refractory to specific epigenetic drugs may be required to improve the therapeutic potential of this approach.

### Effects of LSD1 Inhibitors on the LSD1/GFI1 Complex

GFI1 exerts its function as a transcription factor in part through the recruitment of the histone demethylase LSD1 to target gene promoters via its SNAG domain (Figure 1). LSD1 can remove methyl groups from histone H3 lysine 4 and therefore repress transcription. In specific settings it has been postulated that it can also remove methyl groups from histone H3K9 (76) and subsequently liberate this residue for acetylation, which then causes opening of the chromatin and transcriptional activation (30, 76–79). A number of small molecule LSD1 inhibitors exist and have been used in clinical trials, also as an adjuvant experimental therapy for AML (80). It has been observed that inhibition of LSD1 promotes differentiation of blast cells particularly in cases of AML with chromosomal translocations involving the gene encoding the histone methyl transferase *KMT2A (MLL*) such as the translocation between *KMT2A-MLLT3 (MLL-AF9)* or *KMT2A-MLLT1 (MLL-ENL*). It has been proposed that this differentiation is the consequence of a blockage of LSD1's histone demethylase activity. Indeed, the LSD1 inhibitors NCD25 and NCD38 were found to inhibit growth of MLL-AF9 leukemic cells, but were also active against erythroleukemia, megakaryoblastic leukemia, and MDS cells (30, 77–79). *GFI1* expression was upregulated in this situation and correlated with increased myeloid differentiation. The upregulation of *GFI1* expression in this system coincided also with the activation of so-called super enhancers (SE) one of which was located in the vicinity of the *GFI1* gene locus and with a strong induction of H3K27 acetylation at this SE (77, 79, 81). However, the possibility that LDS1 inhibition simply relieves GFI1 autorepression has also to be considered when interpreting these results. Moreover, although an increase of H3K4 trimethylation was observed upon treatment with the LDS1 inhibitor used in this study, the abundance of H3K4 dimethyl markers decreased contrary to what would have been expected. In patients, a single administration of one of the LSD1 inhibitors (NCD38) caused the elimination of primary MDS-related leukemia cells (81), indicating that this therapeutic route has potential for particular forms of AML and MDS.

**Figure 4:**
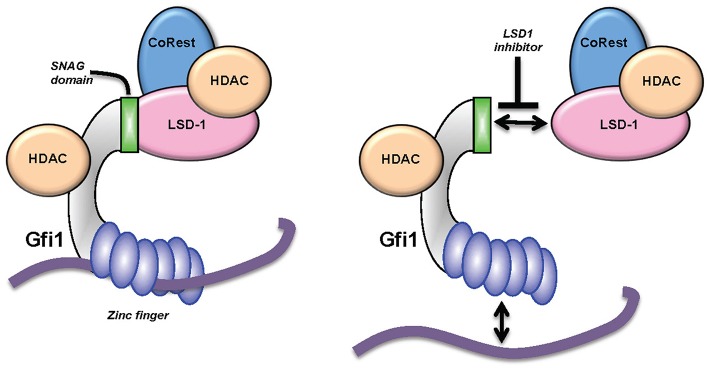
KDM1A (LSD1) inhibitors and GFI1. GFI1 interacts with the histone demethylase LSD1 through its SNAG domain. New data suggest that small molecule inhibitors lead to an eviction of both LSD1 and GFI1 from the DNA (77, 79) and a dissociation of at least part of the HDAC molecules associated with GFI1.

How LDS1 operates on a molecular level under inhibitor treatment remains an open question, in particular since another study revealed that changes in mRNA expression patterns observed after treatment of cells with an LSD1 inhibitor occurred without a genome-wide accumulation of H3K4 methylation at LSD1 binding sites as would have been expected (24, 77–79, 82). Moreover, experiments using a demethylase-defective LSD1 mutant restored AML cells treated with LSD1 inhibitor to the same level as the unmutated, active LSD1 (79). In those experiments, it was observed that the LSD1 inhibitor not only disrupted the interaction between LSD1 and GFI1, but that the treatment also led to the eviction of both GFI1 and LDS1 from genomic sites that both co-occupied (Figure 4). One conclusion of this result was that the function of LSD1, when in a complex with GFI1, is not enzymatic but rather that of a scaffold enabling the recruitment of HDACs, which are themselves responsible for the repression of GFI1 target genes that regulate myeloid differentiation (79). This surprising finding and its conclusions have been corroborated by recent experiments using CRISPR/Cas9 drop out screens which confirm a non-enzymatic role of LSD1 in AML (83). How LSD1 inhibition would affect the non-canonical functions of GFI1 in DNA repair or in regulating the activity of p53 has not yet been addressed, but would provide important information to evaluate the impact that these drug candidates may have on myeloid leukemia or other malignancies.

## Outlook

Recent *in vitro* studies using leukemic cell lines suggest that histone modification associated with less dense DNA packing can positively promote chromosomal rearrangements (84, 85). Additional studies revealed that in the case of human leukemia, chromosomal rearrangements take place preferentially at transcriptionally active sites (86, 87). H3K9 acetylation leads to a less densely packed configuration of the DNA in chromatin and, as a consequence, to increased accessibility of RNA polymerases to initiate and elongate transcription resulting in an activation of gene expression. The examples of the effects of the *GFI136N* variant or of low levels of *GFI1* gene expression show that seemingly small changes in transcription factor status can have consequences that significantly alter the efficacy of drugs targeting epigenetic modifications. Taken together, the results suggest that a precise genetic and epigenetic analysis of leukemic cells may be necessary in the future to choose the right therapy. Moreover, since it has become clear that GFI1 is also involved in DNA repair (33), a high level of *GFI1* gene expression may be linked to therapeutic resistance against those drugs that induce DNA damage. In contrast, experiments with *GFI1* knockout cells have shown that GFI1 deficiency leads to more DNA damage, raising the possibility that interference with *GFI1* gene expression could increase the efficiency of chemotherapy in some instances. The example of GFI1 shows that the status of a single transcription factor can have dramatic effects on chromatin organization, epigenetic parameters, and DNA replication with consequences on the efficiency of therapies and effects on disease outcome. More in depth knowledge about transcription factors and their role in leukemia and AML in particular will be critical to make informed choices of therapeutic regimens, especially with respect to epigenetic-targeted drugs.

## Author Contributions

CK and TM drafted the manuscript and approved it.

### Conflict of Interest Statement

The authors declare that the research was conducted in the absence of any commercial or financial relationships that could be construed as a potential conflict of interest.
